# mSphere of Influence: Engineering Microbes

**DOI:** 10.1128/mSphere.00317-19

**Published:** 2019-06-26

**Authors:** Patrick J. McNamara

**Affiliations:** a Department of Civil, Construction and Environmental Engineering, Marquette University, Milwaukee, Wisconsin, USA

**Keywords:** *Mycobacterium*, disinfection, drinking water, microbial ecology

## Abstract

Patrick J. McNamara works in the field of environmental engineering. In this mSphere of Influence article, he reflects on how the papers “Bacterial community structure in the drinking water microbiome is governed by filtration processes” (A. J. Pinto, C. Xi, and L. Raskin, Environ Sci Technol 46:8851–8859, 2012, https://doi.org/10.1021/es302042t) and “Differential resistance of drinking water bacterial populations to monochloramine disinfection” (T. Chiao, T. M. Clancy, A. Pinto, C. Xi, and L. Raskin, Environ Sci Technol 48:4038–4047, 2014, https://doi.org/10.1021/es4055725) by Lutgarde Raskin and colleagues made an impact on him by providing a foundation for the study of microbial ecology in engineering drinking water treatment plants and drinking water distribution systems.

## Abstract (precis)

Patrick J. McNamara works in the field of environmental engineering. In this mSphere of Influence article, he reflects on how the papers “Bacterial community structure in the drinking water microbiome is governed by filtration processes” (A. J. Pinto, C. Xi, and L.

## COMMENTARY

Should we engineer our drinking water treatment plants to select for specific microbial communities? Should we not decide as a society first if we want to control microbial communities in water that people drink? Wait, are we already doing that? Two papers by Lutgarde Raskin’s research group (“Bacterial community structure in the drinking water microbiome is governed by filtration processes” [1] and “Differential resistance of drinking water bacterial populations to monochloramine disinfection” [2]) have influenced the way that the environmental engineering and science community views and understands the roles, intended or not, of engineered systems on microbial communities in our drinking water. The general question posed by these papers is this: do our engineered disinfection processes in drinking water alter microbial communities? The conclusion, of course, was that they do, and disinfection selects for populations resistant to disinfection. Those papers have had broad impact on environmental engineering and science research. Today, the conclusion that engineering controls alter a microbial community seems obvious, as does the fact that drinking water is home to billions of microbes. And yet, I remember thinking when I was a young graduate student seeing this work presented by Ameet Pinto on a poster at the International Society for Microbial Ecology (ISME) meeting in 2010: “Properly disinfected drinking water has abundant microbial communities? What are you doing branching into this drinking water silo that is so heavily dominated by physical-chemical treatment process researchers?”. The conventional wisdom in our field at the time was that microbiologists who focused on viral or bacterial pathogens in drinking water were looking to remove microbes. These papers by the Raskin group revealed the microbial ecology of drinking water engineering systems in a broader perspective. Moreover, they highlighted that our engineering controls have a substantial impact on the microbes in the water that we drink. While some people might be opposed to the notion of seeding the human gut through drinking water, it was clear that we were ostensibly already doing it.

These papers were influential for more than just the key findings, such as that drinking water treatment might select for *Mycobacterium*. Indeed, the approach that they employed laid robust groundwork for the fundamental study of microbes in engineering systems. There is always a trade-off between absolute control in the laboratory and real-world significance. The authors recognized and addressed this through their robust experimental design. In the monochloramine paper (2), they combined laboratory-scale studies with a full-scale sampling campaign. They also employed both culture-dependent and culture-independent techniques to study drinking water microbiology, and they employed propidium monoazide (PMA) to focus on live cells. Moreover, they employed replicates, a tactic that seems obvious and necessary but that is sometimes dismissed with the argument that replicate analysis is too expensive. In their full-scale paper on the role of rapid sand filters, they sampled at several different times and at several different locations. When choosing to “replicate or lie” (3), the Raskin group replicates to reach statistically supported conclusions, and their robust approach laid the groundwork for research on microbial ecology of drinking water treatment and distribution systems.

That paper influenced my own thinking on multiple levels, including bringing to my attention the microbial ecology of drinking water. In the environmental engineering field, the study of microbial communities was often associated with wastewater whereas water chemistry and physicochemical treatment processes were associated with drinking water. Following the work from the Raskin group, more research results were generated representing the microbial ecology of full-scale and laboratory-scale drinking water systems. The results indicated that engineering decisions impacted drinking water microbial communities, possibly with consequences that we did not desire, such as selecting for *Mycobacterium*. This research pointed out to me that there are many important questions to answer with respect to drinking water microbial ecology. For example, what are the roles of drinking water distribution system materials or corrosion inhibitors in selecting for antibiotic resistance? Answers will involve complementary approaches employing culture-based and culture-independent techniques along with controlled laboratory-scale experiments as well as real-world sampling campaigns.

The Raskin group has continued this research, more recently focusing on developing novel methods to track *Mycobacterium* (4), and Pinto now has his own group that is also a leader in the microbial ecology drinking water field (5). Following the earlier work from the Raskin group, more research results were generated on full-scale and laboratory-scale drinking water treatment and drinking water distribution systems. For example, Gomez-Smith et al. published on microbial communities in full-scale cement-lined and iron pipes (6). Ji et al. published laboratory-scale studies related to how pipe material impacts microbial community (7), and Garner et al. published full-scale studies on how water reuse impacts antibiotic resistance (8). The number of citations of the early drinking water papers published by the Raskin group indicates the instant classic nature of their research. An environmental engineer who is a microbial ecologist is no longer assumed to be in either bioremediation or wastewater. Drinking water microbial ecologists emerged from their siloed pipes and now belong to a more fully acknowledged, important research field working to protect public health in broader ways.
